
JOURNAL INFORMATION
==============================
NLM Title Abbreviation: ScientificWorldJournal
Journal ID: ScientificWorldJournal
NLM Journal ID: 101131163
Journal ID: TSWJ

The Scientific World Journal

ISSN: 2356-6140
EISSN: 1537-744X
Publisher: Wiley

ARTICLE INFORMATION
==============================
PMCID: PMC6134968
PMID: 30245597
DOI: 10.1100/tsw.2001.133
Article ID: 120916
Article version: 1
Subjects: Short Report

Role of Mitochondrial DNA in Aging Processes

Attardi Giuseppe M.
California Institute of Technology, Pasadena, CA 91125, USA
Publication date: 2001
Electronic publication date: 2001 Nov 3
Volume: 1
First page: 76
Last page: 76
Copyright: Copyright © 2001 Giuseppe M. Attardi.
Copyright year: 2001
License: This is an open access article distributed under the Creative Commons Attribution License, which permits unrestricted use, distribution, and reproduction in any medium, provided the original work is properly cited.
License URL: https://creativecommons.org/licenses/by/3.0/

==============================
Miami *Nature Biotechnology* Short Reports
*TheScientificWorld* (2001) 1(S3), 76SR
ISSN 1532-2246; DOI 10.1100/TSW.2001.133

### ROLE OF MITOCHONDRIAL DNA IN AGING PROCESSES

Giuseppe M. Attardi

California Institute of Technology, Pasadena, CA 91125, USA
Attardig@seqaxp.bio.caltech.edu

The idea that during mammalian cell aging there is an increasing incidence of mitochondrial DNA (mtDNA) mutations has received, in the last ten years, support from direct experimental observations of an aging-related occurrence in this DNA of oxidative and alkylation derivatives of nucleotides, of small deletions and insertions and, especially, of large deletions. However, the low frequency of these mutations has raised questions about their functional significance. Furthermore, in contrast to the large deletions, the search for aging-dependent point mutations in human mtDNA has given inconclusive and often discordant results. This is in part due to the lack of a sensitive and reliable method for detecting mutations which occur in heteroplasmic form, i.e., together with wild-type mtDNA, as expected for aging-related mutations, and to the search having been largely limited to the protein- and RNA-coding regions of mtDNA. It is known that the main control region of mtDNA, that includes the D-loop and adjacent transcription promoters, is the most variable portion of the human mitochondrial genome, in which heteroplasmic point mutations have been reported, and for which there may be a higher probability of aging-related damage. In the present work, for the reasons stated above, the main mtDNA control region, in particular, the segment that contains the critical control sequences for mtDNA replication, was chosen for a detailed analysis of aging-dependent mtDNA damage. For this purpose, a novel approach for the specific detection of heteroplasmic mtDNA mutations, in particular, one that also excluded any interference by nuclear mtDNA pseudogenes, was developed. The use of this approach surprisingly revealed high copy point mutations at specific positions in the control region of human fibroblast mtDNA from normal old, but not young individuals. Furthermore, longitudinal studies showed the appearance of the mutations in a given individual only at advanced age. Most dramatically, some mutations appeared in more than one individual. Thus, a T to G transversion at position 414 was found, in a generally high proportion (up to 50%) of mtDNA molecules, in eight of 14 genetically unrelated individuals above 65 years of age (57%), while it was not detected in any of 13 individuals younger than 65. These observations clearly pointed to a novel phenomenon, and provided the first evidence of a large accumulation of aging-dependent point mutations in mtDNA.

With the aim of understanding the mechanism leading to the appearance of the aging-dependent large accumulation of specific point mutations at critical sites of the control region of human mtDNA, fibroblast cultures carrying a high proportion of the most common of these mutations, i.e., the T414G transversion within the promoter for the primer of heavy mtDNA strand synthesis, were subcloned. In other experiments, the mutation was transferred to mtDNA-less cells by mitochondria-mediated transformation. These experiments revealed the clonal nature of the phenomenon being investigated. Thus, a significant fraction of the original fibroblasts and of the cells of the derived transmitochondrial cell lines were found to carry the mutation in a very high proportion of their mtDNA, up to a homoplasmic form. These findings pointed to an intracellular replicative advantage of the mutant molecules, and to either multiple independent initiating events or a growth advantage of the mutant cells. Furthermore, long-term *in vitro* culture of fibroblasts carrying the mutation from three different individuals showed a clear tendency of the cultures to lose the mutation within two or three months, although at different rates. Fibroblast cultures from three individuals lacking the mutation failed to acquire it spontaneously during the same period. These experiments indicated that the observed dramatic expansion of the mutations requires an *in vivo* specific environment.

In order to establish the generality of the phenomenon under investigation, a search was carried out for the accumulation of specific point mutations in the mtDNA control region of a typical postmitotic tissue, i.e., skeletal muscle. This search yielded positive results, revealing, however, an unexpected tissue specificity of such mutations. Thus, muscle from older human individuals exhibited an accumulation of specific point mutations in the mtDNA control region, which have not been found in the corresponding mtDNA region from fibroblasts of older subjects. Similarly, the site-specific point mutations, which accumulate with aging in the fibroblast mtDNA control region, have not been detected, or only at marginal levels, in muscle mtDNA. Such a striking tissue specificity of aging-related mtDNA mutations in critical sites for mtDNA replication strongly points to their functional relevance.
